# The chronology of reindeer hunting on Norway's highest ice patches

**DOI:** 10.1098/rsos.171738

**Published:** 2018-01-24

**Authors:** Lars Pilø, Espen Finstad, Christopher Bronk Ramsey, Julian Robert Post Martinsen, Atle Nesje, Brit Solli, Vivian Wangen, Martin Callanan, James H. Barrett

**Affiliations:** 1 Department of Cultural Heritage, Oppland County Council, PO Box 988, 2626 Lillehammer, Norway; 2 School of Archaeology, University of Oxford, 1 South Parks Road, Oxford OX1 3TG, UK; 3 Museum of Cultural History, University of Oslo, PO Box 6762, St. Olavs plass, 0130 Oslo, Norway; 4 Department of Earth Science, University of Bergen, PO Box 7803, 5020 Bergen, Norway; 5 Department of Historical Studies, NTNU Norwegian University of Science and Technology, Trondheim, Norway; 6 McDonald Institute for Archaeological Research, University of Cambridge, Downing Street, Cambridge CB2 3ER, UK

**Keywords:** reindeer hunting, climate change, economic intensification, glacial archaeology, alpine travel, historical ecology

## Abstract

The melting of perennial ice patches globally is uncovering a fragile record of alpine activity, especially hunting and the use of mountain passes. When rescued by systematic fieldwork (glacial archaeology), this evidence opens an unprecedented window on the chronology of high-elevation activity. Recent research in Jotunheimen and surrounding mountain areas of Norway has recovered over 2000 finds—many associated with reindeer hunting (e.g. arrows). We report the radiocarbon dates of 153 objects and use a kernel density estimation (KDE) method to determine the distribution of dated events from *ca* 4000 BCE to the present. Interpreted in light of shifting environmental, preservation and socio-economic factors, these new data show counterintuitive trends in the intensity of reindeer hunting and other high-elevation activity. Cold temperatures may sometimes have kept humans from Norway's highest elevations, as expected based on accessibility, exposure and reindeer distributions. In times of increasing demand for mountain resources, however, activity probably continued in the face of adverse or variable climatic conditions. The use of KDE modelling makes it possible to observe this patterning without the spurious effects of noise introduced by the discrete nature of the finds and the radiocarbon calibration process.

## 1. Introduction

In the context of global warming [1], the melting of perennial ice patches is revealing and destroying an unprecedented archaeological record of high-altitude hunting and the use of mountain passes [2–6]. In response, a new research subject, glacial archaeology, is rapidly developing to rescue now-threatened artefacts and to study the relationship between variability in climate and the intensity of human use of alpine landscapes. Jotunheimen and surrounding mountain areas of Oppland, with Norway's highest mountains (to 2469 m), are a key focus of this global research [7–11]. Here systematic survey of contracting ice-edges has recovered emerging artefacts of wood, textile, hide and other organic materials that are otherwise rarely preserved. By radiocarbon dating 153 of these finds, and considering their taphonomic, environmental and socio-economic context, we reveal trends in the relationship between climate and the intensity of reindeer hunting and other high-alpine activities from *ca* 4000 BCE to the present. A new kernel density estimation (KDE) method is used to determine the distribution of radiocarbon dated events without the confounding effects of spurious peaks from the shape of the radiocarbon calibration curve [12]. Thus it is possible to evaluate the relationship between the chronology of ice-patch finds and key thresholds. These include both environmental turning points such as the onset of mid-Holocene neoglaciation, the Late Antique Little Ice Age and the Little Ice Age—and episodes of cultural transformation such as the spread of permanent agricultural settlement and the growth of long range intra- and inter-regional trade which may have reached an apogee during the Viking Age.

Variations in the intensity of activity in mountainous environments globally are often linked to climate change. Sometimes the mechanism is as straightforward as access, with fluctuating glaciers blocking or clearing alpine passes [13]. In other contexts environmental change is thought to affect regional demographic patterns, with population pressure then influencing the use of high-elevation areas [14,15]. Alternatively, climate has been interpreted as a mediator of alpine resource distributions, drawing game animals such as reindeer, bighorn sheep and vicuña (and thus their hunters) to varying elevations [4,7,16,17]. In a Norwegian context, episodically colder temperatures may for example have made reindeer available to ice-patch hunters at lower elevations (below the zone of surviving archaeological ice-patch finds) [7].

Alternatively, increasing use of high elevation resources is sometimes interpreted as a matter of economic intensification and ‘globalization’, only indirectly influenced by climate. In times of demographic and/or economic expansion, mountains, like oceans and forests, may become part of an expanding ecological frontier serving the needs of both local settlements and distant (often urban) consumers linked via long-range exchange networks [18–20]. In Norway, increases in mountain hunting and travel (including transhumance [21]) have been associated with changing demands on resources. In particular, the intensification of reindeer hunting has been attributed to the growing trade of fur and antler (the latter of which was used for artefacts such as combs) during the Viking Age (800–1050 CE) and Middle Ages (1050–1537 CE) [22–28]. These products met the demands of both regional central places (towns by the end of the Viking Age) and export. Transport of reindeer antler to Denmark is now documented as early as the eighth century CE [29]. The first systematic records of reindeer pelt exports (to England) do not appear until the early fourteenth century CE, but they illustrate a trade that may have started before the existence of relevant historical documentation [30]. It has been suggested that Norwegian reindeer pelts and antlers may even have been exported as early as the Roman Iron Age (1–400 CE) [31].

To evaluate these alternative interpretations, which need not be mutually exclusive, it is necessary to compare archaeological evidence regarding the chronology of mountain hunting and travel with concurrent climate changes. Were variations in the abundance of ice-patch finds associated with or independent of warming and cooling, shrinking ice or growing ice? Were efflorescences of ice-patch hunting concurrent with or unrelated to periods of external demand for reindeer antler and/or fur? The new radiocarbon dated finds from Norway's ice patches reported here, combined with KDE analysis, provide a unique opportunity to address these questions. They can illuminate the interaction of climatic and socio-economic factors in an alpine context where all pre-industrial human activity is potentially susceptible to environmental limits set by accessibility, exposure and resource availability.

## 2. Material and methods

**Figure 1.**
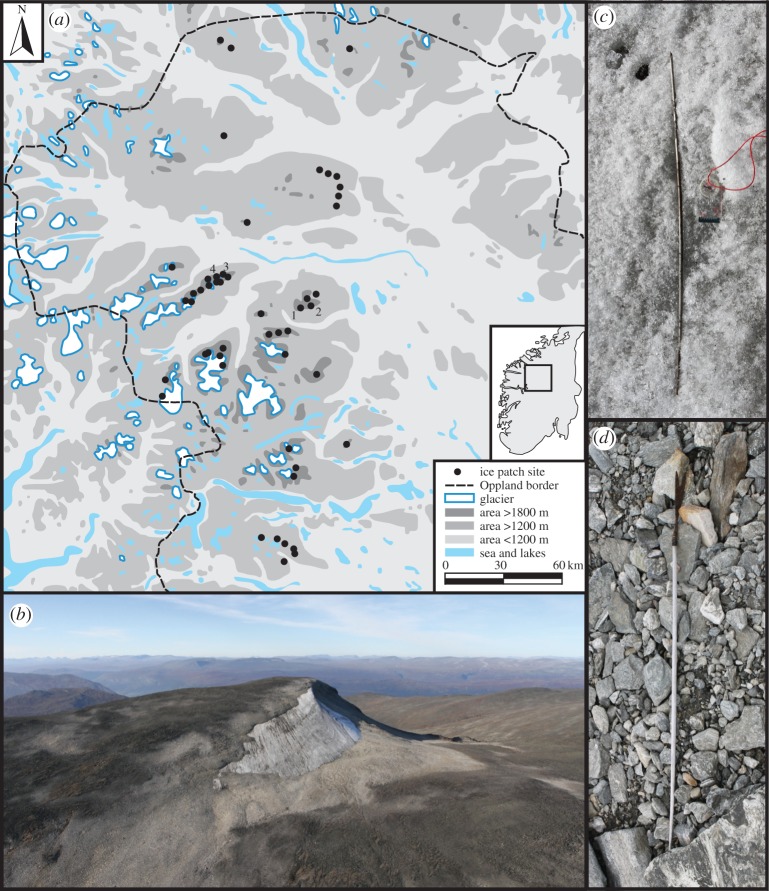
The melting of perennial ice patches in Oppland, Norway, is yielding numerous organic artefacts. (*a*) Distribution map of archaeological sites at glaciers and ice patches in Oppland. Ice patches mentioned in text: 1, Storfonne; 2, Langfonne; 3, Åndfonne; 4, Lendbreen. (*b*) A 2014 aerial photograph of the Storfonne ice patch, from the south. The light coloured areas (lichen free zones) have recently been exposed by the melting ice patch. (*c*) A 92 cm arrow shaft (C59804/41) from the Langfonne ice patch, dated to 2031–1879 BCE (UBA-29712, calibrated at 95% probability). (*d*) An 88 cm arrow (C57838/14(249)) from the Åndfonne ice patch, with iron arrowhead, sinew, and wooden shaft, dated to 695–891 CE (Beta-320917, calibrated at 95% probability).

Over 2000 artefacts and 375 samples of associated materials (e.g. reindeer antlers and bones, horse bones and concentrations of horse dung) have been recovered from Oppland's mountain ice between 2006 and 2015. Most are from stationary (negligible basal movement) ice patches rather than glaciers, because the continuous movement of the latter destroys objects at the base of the ice [32]. Perennial ice patches yielding artefacts mostly occur over 1400 m, with only shaded canyon examples being known from lower elevations (figure 1). The ice patches are situated above the treeline in areas of blockfields, bouldery till, scree and bedrock, which remain snow-covered for nine or more months a year. Ice patches expand and contract through time. Annual growth occurs under conditions of high winter snowfall, high winter drifting and/or low summer temperature. Conversely, negative mass balance happens if there is little snow accumulation in winter and/or summer conditions are warm [33].

**Figure 2.**
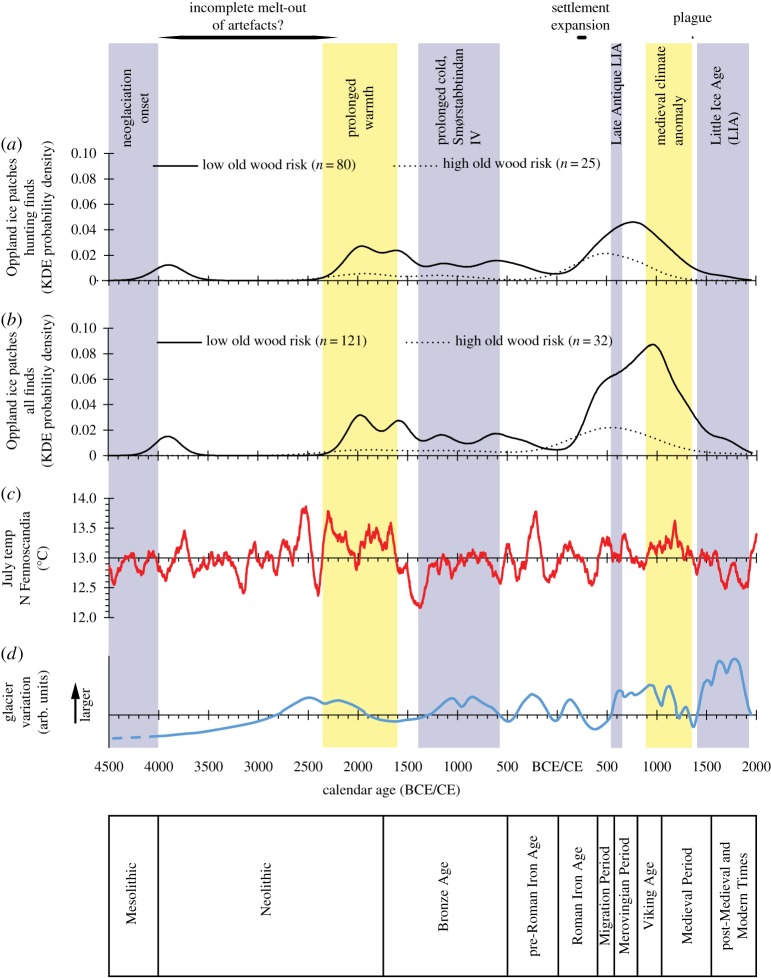
Long-term climate fluctuations and the chronological distribution of ice-patch finds from Oppland, Norway. (*a*) The mean kernel density model distributions of radiocarbon dates on artefacts from ice patches in Oppland that are associated with hunting. Objects at high risk of an old wood effect, making them appear older than the date of deposition, have been plotted separately. (*b*) The mean kernel density model distributions of radiocarbon dates on all artefacts from ice patches in Oppland. Objects at high risk of an old wood effect, making them appear older than the date of deposition, have been plotted separately. (*c*) Estimated July temperature (°C) in northern Fennoscandia based on tree-ring width data [32,37]. The *x*- and *y*-axes intersect at mean temperature between 4500 BCE and 2000 CE. (*d*) Relative glacier size in the Smørstabbtindan massif, Jotunheimen, between 4500 BCE and 1950 [38]. The *x*- and *y*-axes intersect at modern glacier size.

Cold climate conditions in Scandinavia can be associated with either increased or decreased likelihood of precipitation [34]. Thus there is not a direct relationship between temperature fluctuations and the extent of high-mountain ice. To interpret the chronological distribution of ice-patch finds one has to consider both variables. The most relevant continuous temperature proxy for the chronological range of Norwegian ice-patch finds is that of Helama *et al*. [35], based on tree-ring width data from across northern Fennoscandia. Other alternatives are discontinuous [36], of shorter duration [34,37] and/or use distant proxies such as tree-ring chronologies from the Russian Altai and European Alps [38]. Figure 2 includes reconstructed average July temperature from 4500 BCE to 2000 CE [39], smoothed using a 100-year running mean. There is no independent long-term record of fluctuations in the size of Norwegian perennial ice patches. However, a Holocene glacier curve has been established for the Jotunheimen area based on study of the Smørstabbtindan massif [40]. Glaciers and stationary ice patches share the same influences on mass balance, accepting that the latter may be more influenced by snow drifting (due to wind conditions during the accumulation season) on an inter-annual scale [33]. Figure 2 includes the Smørstabbtindan glacier curve from 4500 BCE to 1950 CE.

The radiocarbon dates reported here are exclusively on materials that reached the high mountains due to the activities of people. Entirely natural samples of *in situ* vegetation, shed antlers and reindeer dung are omitted. The dated finds range from manufactured objects (e.g. arrows) to incidental traces of human journeys (e.g. horse dung). The dates have been obtained from 23 discrete ice patches, a subset of the 51 presently known artefact-yielding examples in the high mountains of northern Oppland County (figure 1).

The dated objects fall into two broad categories. Most are associated with hunting. In Norwegian mountains, reindeer frequently congregate on ice patches in July and August in order to avoid parasitic insects [41]. Hunters clearly followed, occasionally losing arrows and scaring sticks (poles, sometimes with moving flags of wood or bark, probably set up in rows to direct the movement of deer towards waiting archers). Objects less easily lost, such as bows, were sometimes discarded if broken. A second category of artefacts is more diverse (e.g. tools, skis, clothing, rags, horse gear, horse bones and horse dung) and may represent hunting or mountain travel for other purposes. Thus the radiocarbon dates are grouped either as hunting finds or as all finds combined for the purposes of interpretation (figure 2).

The find location, object type, raw material and radiocarbon dating information for each dated object is provided in the electronic supplementary material, table S1. Where practicable, the dates are on materials likely to have a short inherent age. However, most ice-patch finds are of wood, including long-lived taxa such as ash (*Fraxinus excelsior*), elm (*Ulmus glabra*), willow (*Salix* spp.) and especially Scots pine (*Pinus sylvestris*)—the latter of which can live to between 200 and 677 years in Norway based on dendrochronological research (T Thun 2017, personal communication). Thus there is a high risk of old wood effect, in which radiocarbon dates are earlier than the time-period of artefact deposition. Here we divide the artefacts into groups with low and high risk of an old wood effect. The low risk materials include wool, antler, animal hide, horse dung and wood of the comparatively short-lived trees alder (*Alnus* spp.) and birch (*Betula pubescens*). Most of the arrows and many other finds are made of birch which is unlikely to exceed an age of *ca* 100 years [42]. Individual artefacts clearly made from thin branches (e.g. some arrows and scaring sticks) also have a low risk of high inherent age, regardless of wood species. Conversely, objects from split timbers of ash, elm, willow and pine have a high risk of being old wood.

Summed probability distributions of radiocarbon dates are widely used to infer the intensity of past human activity, demographic trends and their relationship with climate change [4,14,43–45]. They can be misleading, however, due to calibration ‘noise’ (both peaks and troughs introduced by the shape of the ^14^C calibration curve) [12]. Various approaches have been attempted to circumvent calibration noise, with simulation-based methods among the most common. The latter are effective at determining significant peaks in the summed probability of radiocarbon dates when sample sizes are large. However, they entail a high risk of type II errors (failing to detect real patterns) when the number of dates per category is small, as here [46].

We use a new statistical method based on kernel density estimation (KDE) [12]. This takes as its starting point the likelihood distributions generated by calibration of the radiocarbon analyses against IntCal13 [47]. The model employed assumes that the distribution of dated events can be well represented by a KDE distribution. The procedure involves the use of a Markov chain Monte Carlo (MCMC) and for each pass of the model the KDE for the distributions is used as an additional term in the likelihood for each event as well as being used to summarize the distribution. The average of the KDE distributions generated during the MCMC process is taken to be the best estimate of the underlying distribution of events and the degree of variability in these distributions can also be assessed to see how robust this estimate is (see for example electronic supplementary material, figure S1). In practice this approach can be shown [12] to eliminate both the noise generated by the calibration process (usually seen in sum distributions) and the excessive spread in data seen with unmodelled data. The results of kernel density models and summed probability distributions are compared in the electronic supplementary material, figure S1. Interpretations are based on KDE in the text except where noted otherwise.

## 3. Results

The earliest isolated ice-patch finds are from after the initial onset of Holocene neoglaciation *ca* 4000 BCE. A subsequent gap exists between *ca* 3800 and *ca* 2200 BCE, over which time temperature and glaciation were variable. Ice-patch finds in this date range are known from elsewhere in Norway, but they are very rare [48,49]. In Oppland there are late Neolithic/early Bronze Age and Viking-age peaks in artefact chronology, associated with warm periods with or without large glaciers (see figure 2 for the absolute chronology of archaeological periods). Conversely, prolonged cold periods with large glaciers in both the late Bronze Age (the Smørstabbtindan IV glacier maximum) and late medieval/post-medieval times (the Little Ice Age) are associated with a low KDE for dated objects.

The years of the sequential Roman Iron Age, Migration Period, Merovingian Period and early Viking Age reveal a more complex relationship between artefact chronology and climate (figure 2). Here the KDE rises rapidly in a time of cooling and shrinking glaciers, continues into a time of warmth and growing glaciers, and remains high even in the Late Antique Little Age (LALIA) of 536 to *ca* 660 CE, when rapid cooling brought on by volcanism and sustained by a solar minimum is argued to have led to major societal collapses in Scandinavia and globally [50–53].

Based on objects with a low risk of old wood effect, the KDE probability continues to rise in the Merovingian Period, reaching a peak in the Viking Age before declining in the Middle Ages. The chronology of hunting and all finds diverges slightly towards the end of the first millennium CE. Hunting equipment peaks in the eighth to ninth centuries CE whereas the combined distribution of dates on all finds peaks later, in the tenth century. This difference is caused by a cluster of artefacts from the mountain pass site of Lendbreen that date to late in the Viking Age. Many of the latter may relate to intra-regional travel (perhaps transhumance) rather than reindeer hunting.

The KDE distributions based on objects with a high risk of old wood effect demonstrate similar overall patterns, but with a variable offset in absolute chronology as one would expect given the use of raw materials of differing inherent age (figure 2). The ‘high risk’ hunting-related materials exhibit the most striking difference between the kernel density models and conventional summed probability plots (electronic supplementary material, figure S1). The summed probability plots for these finds show a trough in the Merovingian Period that is not reflected in the kernel density distributions derived from the same radiocarbon dates. This trough is also reflected in the summed probability plot of all dates with a high risk of old wood (hunting-related finds provided 25 of the total of 32 relevant samples).

## 4. Discussion

The earliest dates on ice-patch finds are likely to reflect preservation factors. Before neoglaciation began *ca* 4000 BCE, during the high temperatures of the early Holocene, there may have been little ice in the Oppland mountains in which artefacts could be preserved [40,54].

A subsequent gap between *ca* 3800 and *ca* 2200 BCE may exist because the oldest finds have been lost to ancient melting at low elevations, and are still trapped in ice at high elevations. No finds older than 3610 ± 30 uncalibrated BP (2110–1889 BCE at 95.4% probability) were found at elevations under *ca* 1900 m (electronic supplementary material, figure S2). Thus it is reasonable to suggest that lower-elevation ice patches have melted periodically prior to this date, leading to the destruction of the artefacts they contained. Conversely, at elevations higher than *ca* 1900 m the only finds older than this (*ca* 2000 BCE) are from southerly facing exposures. At these elevations it is probable that the oldest finds, in the oldest and deepest ice, have not yet melted out from sites exposed to less summer sun.

During the late Neolithic/early Bronze Age it is plausible that warm conditions were favourable for hunting at ice patches—with neither concurrent nor subsequent episodes of melting being severe enough to obliterate the evidence. In the late Bronze Age and during the Little Ice Age (of late medieval and post-medieval times), conversely, cold conditions may have been less favourable for high-mountain activities, be it due to direct impacts on accessibility and reindeer distributions [7] or indirect effects on the scale of demand for mountain products. Such indirect effects might result from declines in population and/or the degree of inter-regional exchange.

This interpretation, that there was a positive association between temperature and high-elevation activity, is plausible as a generalization. However, it must be qualified based on other archaeological and historical evidence. Most intriguing are times when ice-patch finds increased during periods of cooling. These are explicable in light of regional and supra-regional archaeological evidence regarding settlement, transhumance, exchange and other mobility. The rapid increase in the number of dated artefacts around the third century CE is contemporary with archaeological evidence from rescue excavation and pollen analysis for expansion of permanent agricultural settlement in the mountain valleys of Oppland [10,55,56]. With settlement expansion, the potential and necessity to carry out high-elevation hunting and to use mountain passes can only have increased. It is not yet possible, however, to evaluate whether reindeer pelts and antlers were exported at this date. Contemporary historical or archaeological evidence of reindeer products from potential consumer regions within the Roman Empire remains to be found [57].

The continuing rise in ice-patch finds during the LALIA, at the transition from the Migration to Merovingian Periods, is surprising given low temperatures. Yet arguably the importance of hunting could have peaked when yields from traditional agriculture would have been low. Population is thought to have dropped across Norway and Scandinavia during and after the LALIA, based on a decrease in the number of dated graves and settlement sites [51,58]. This wider pattern is not reflected in the KDE plots of the ice-patch data (figure 2), nor in traditional summed probability plots of the finds with a low risk of old wood effect (electronic supplementary material, figure S1).

There is a ‘Merovingian trough’ in the summed probability plots of the radiocarbon dates on objects with a high risk of an old wood effect (electronic supplementary material, figure S1). Three factors combine to create this discrepancy with the KDE model. Firstly, the peak of ‘old wood’ finds from the Roman Iron Age and Migration Period is enhanced by a time-limited fashion for the manufacture and use of composite scaring sticks (with separate staff and flag) of split pine. Scaring sticks of this date range are thus more frequent among the ‘high risk’ than the ‘low risk’ finds, contributing to a mode in the Roman Iron Age/Migration Period only for the former of these datasets. Secondly, the total number of ‘high risk’ dates [32] is not large and the KDE takes into account the fact that a lack of data might simply be due to chance and so is not sensitive to short-lived fluctuations; one of the key features of the KDE model, or indeed of KDE generally, is to evaluate the significance of the number of sampled events within a distribution. Thirdly, the KDE model estimate for this period has quite a wide uncertainty indicating that there is insufficient information to determine whether or not there is a significant decline in this period (electronic supplementary material, figure S1). In summary, it is not yet possible to say with confidence whether or not there was a brief decline in mountain activity in Oppland following the LALIA. It does seem clear, however, that any such downturn or hiatus was short-lived. The model of widespread societal and demographic collapse in sixth-century Scandinavia may thus need to be refined, although the likelihood that mountain hunting would continue or increase (to supplement declining agricultural productivity) in times of cooling must also be recognized.

Burials and farms became frequent again in Norway during the Viking Age [58]. This internal colonization, echoed in the efflorescence of overseas Scandinavian migration and trade known as the Viking Age, may explain the eighth--ninth- and tenth-century peaks in the abundance of hunting and all ice-patch finds respectively. There was more long-distance mobility [59,60], more demand for mountain products such as furs and antlers [29] and perhaps more people to feed by transhumance. It is likely to be relevant that in the years around 1000 CE the process of urbanization in Norway and elsewhere in Europe created new markets for trade in products from the mountains and forests [10,23]. Other extensive methods of resource extraction, such as sea fishing, were increasing around the North Sea region at the same time [61,62]. All of this activity reached its apogee concurrently with the Medieval Climate Anomaly (also known as the Medieval Warm Period), but the rise in ice-patch activity began earlier and, as we have shown, continued through varying environmental conditions.

The Viking-age peak in ice-patch finds waned before the onset of the Little Ice Age. One contributing factor was probably the replacement of bow hunting for reindeer with mass-harvesting techniques [10,11,22]. The latter methods included large pitfall trapping systems, funnel-shaped trapping systems and water-trapping systems incorporating natural lakes [22,23,63,64]. Use of these intensive and extensive systems peaked in the thirteenth century, which may have reduced wild reindeer populations [23,65]. As the Little Ice Age set in the Norwegian population was concurrently affected by plague, first striking in 1349–1350 CE [66,67], which must have led to a reduction in both the demand for mountain products and the available labour to acquire them. The demographic crisis affected society severely. Trade and markets decreased, marginal farms were abandoned and even the degree of urbanization was affected—with smaller towns like Borgund, Veøy and Kaupanger falling into disuse [68]. Finally, the introduction and more widespread use of firearms during the Little Ice Age, probably from the seventeenth century onwards, reduced the likelihood of ice-patch losses and finds even when hunting was practised.

## 5. Conclusion

The varying abundance of surviving and dated artefacts from Oppland's ice patches reveals the complex and sometimes counterintuitive interaction of climate change, preservation conditions, land-use history and both local and external socio-economic drivers. Hunters and farmers used their knowledge of the mountains in diverse and changing circumstances. Cold temperatures may sometimes have kept them from Norway's highest elevations, as one might expect based on factors such as accessibility, exposure and game distributions [7]. In times of high socio-economic demand or probable societal stress, however, mountain activity may have continued despite adverse or variable climatic conditions. Crucially, the use of KDE modelling makes it possible to observe this patterning without the spurious effects of noise introduced by the discrete nature of the finds and the radiocarbon calibration process. Moreover, our resulting observations may provide one explanation of the often poor association between ice-patch finds and climatic trends globally [2]. Given that there are now over 2000 artefacts from Oppland's ice patches, the Norwegian evidence offers an ongoing opportunity to explore long-term interaction of environmental, demographic and socio-economic change. The 153 radiocarbon dates discussed here have already illuminated varying human--environment interaction in a context highly susceptible to the influence of climate change, while concurrently highlighting the importance of an increasingly threatened source of archaeological and palaeoenvironmental evidence.

## Supplementary Material

Figure S1

## Supplementary Material

Figure S2

## Supplementary Material

Table S1

## Data accessibility

The electronic supplementary material includes table S1, Radiocarbon dates and associated data for artefacts from ice patches in Oppland.
