# Supplementary figures and images for: The chronology of reindeer hunting on Norway's highest ice patches
Source: R Soc Open Sci. 2018 Jan 24;5(1):171738. doi: 10.1098/rsos.171738 (PMC5792946; doi:10.1098/rsos.171738)

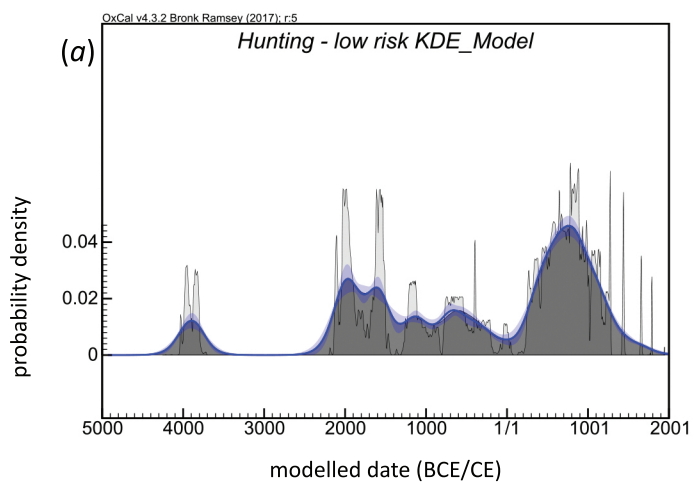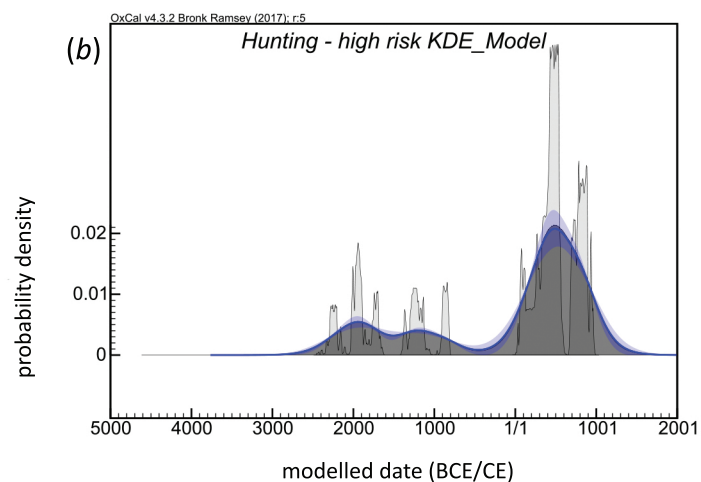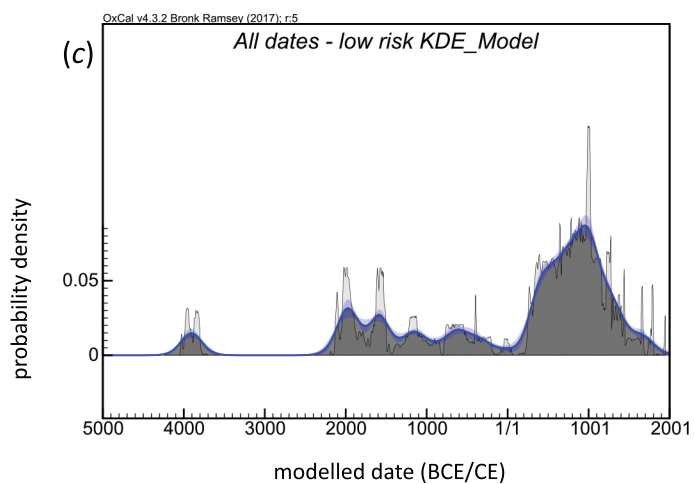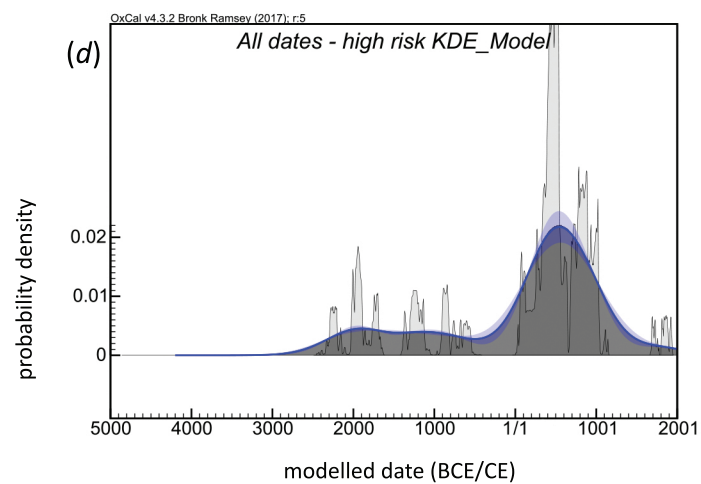

Supplement: Figure S1 [file rsos171738supp1.pdf]

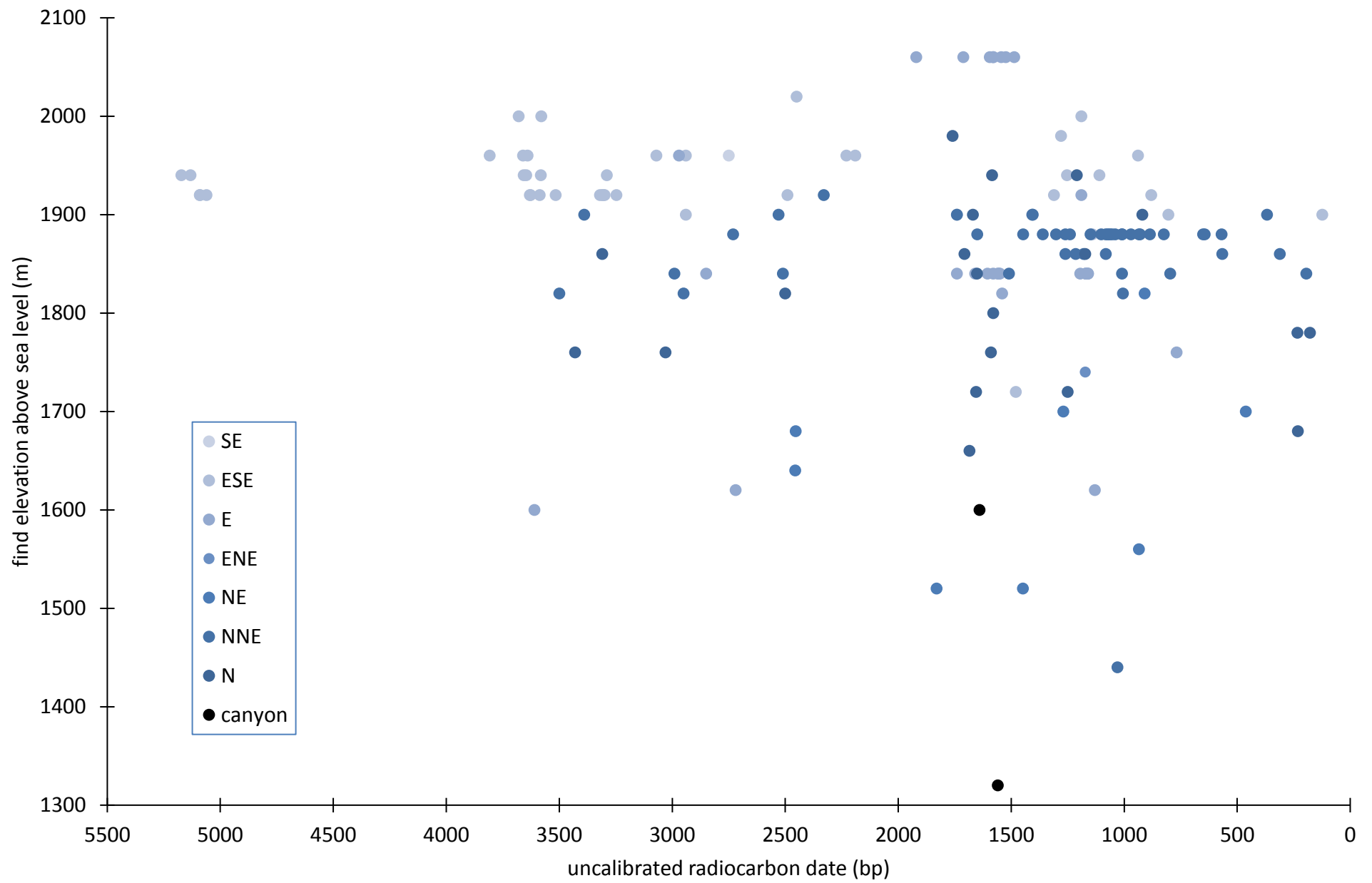

Supplement: Figure S2 [file rsos171738supp2.pdf]
